# Detecting changes in the performance of a clinical machine learning tool over time

**DOI:** 10.1016/j.ebiom.2023.104823

**Published:** 2023-10-02

**Authors:** Michiel Schinkel, Anneroos W. Boerman, Ketan Paranjape, W. Joost Wiersinga, Prabath W.B. Nanayakkara

**Affiliations:** a Center for Experimental and Molecular Medicine (CEMM), Amsterdam UMC, University of Amsterdam, Amsterdam, the Netherlands; b Division of Acute Medicine, Department of Internal Medicine, Amsterdam UMC, VU University, Amsterdam, the Netherlands; c Department of Clinical Chemistry, Amsterdam UMC, VU University, Amsterdam, the Netherlands; d Division of Infectious Diseases, Department of Internal Medicine, Amsterdam UMC, University of Amsterdam, Amsterdam, the Netherlands

**Keywords:** Machine learning, Performance drift, Artificial intelligence, Statistical process control, Validation

## Abstract

**Background:**

Excessive use of blood cultures (BCs) in Emergency Departments (EDs) results in low yields and high contamination rates, associated with increased antibiotic use and unnecessary diagnostics. Our team previously developed and validated a machine learning model to predict BC outcomes and enhance diagnostic stewardship. While the model showed promising initial results, concerns over performance drift due to evolving patient demographics, clinical practices, and outcome rates warrant continual monitoring and evaluation of such models.

**Methods:**

A real-time evaluation of the model's performance was conducted between October 2021 and September 2022. The model was integrated into Amsterdam UMC’s Electronic Health Record system, predicting BC outcomes for all adult patients with BC draws in real time. The model’s performance was assessed monthly using metrics including the Area Under the Curve (AUC), Area Under the Precision-Recall Curve (AUPRC), and Brier scores. Statistical Process Control (SPC) charts were used to monitor variation over time.

**Findings:**

Across 3.035 unique adult patient visits, the model achieved an average AUC of 0.78, AUPRC of 0.41, and a Brier score of 0.10 for predicting the outcome of BCs drawn in the ED. While specific population characteristics changed over time, no statistical points outside the statistical control range were detected in the AUC, AUPRC, and Brier scores, indicating stable model performance. The average BC positivity rate during the study period was 13.4%.

**Interpretation:**

Despite significant changes in clinical practice, our BC stewardship tool exhibited stable performance, suggesting its robustness to changing environments. Using SPC charts for various metrics enables simple and effective monitoring of potential performance drift. The assessment of the variation of outcome rates and population changes may guide the specific interventions, such as intercept correction or recalibration, that may be needed to maintain a stable model performance over time. This study suggested no need to recalibrate or correct our BC stewardship tool.

**Funding:**

No funding to disclose.

**Research in context**

**Evidence before this study**

A thorough PubMed title/abstract search was conducted from inception to May 19, 2023, using the terms “Machine Learning” OR “Prediction” AND “Performance drift” OR “Performance monitoring” OR “Statistical process control”. This search produced 157 unique articles, supplemented by additional relevant studies found within their references. Existing research on evaluating performance drift in clinical prediction tools is sparse. The focus of these studies predominantly lies in retraining existing models and assessing improvements in their performance, rather than initially identifying whether performance drift exists. A few of these studies have attempted to monitor performance over time, with a preference for interrupted time-series analyses.

**Added value of this study**

This study presents a robust and systematic approach to monitoring the performance of clinical prediction models using Statistical Process Control charts. By tracking both the model's performance and population characteristics, such as outcome rates, it offers a straightforward approach to detect changes in predictions that exceed expected variation.

**Implications of all the available evidence**

The paucity of literature on the continuous monitoring of performance drift implies that this crucial aspect of deploying clinical machine learning models is often neglected. Despite some attempts to tackle this issue documented in the literature, these methods lack simplicity for integration into routine practice. This study emphasizes the importance of monitoring performance drift and presents an easily applicable method to do so. It sets the stage for further research in this direction, promoting the reliable and robust application of machine learning models in healthcare settings.

## Introduction

Excessive utilization of blood cultures (BCs) in Emergency Departments (EDs) results in low yields and high contamination rates, contributing to increased antibiotic usage, downstream diagnostics, and prolonged hospitalization.^1^, ^2^, ^3^ Recently, we developed and shared a machine learning model for BC stewardship in *eBioMedicine*.^4^ The tool is meant to provide stewardship to dissuade physicians from ordering blood cultures for patients whom the physicians would already be inclined to perform blood cultures for. By utilizing laboratory results and vital signs, the model reached Areas Under the Curve (AUCs) between 0.75 and 0.81 for predicting BC outcomes in various ED cohorts. The tool identifies patients at low risk of bacteremia, in whom BC analyses may safely be avoided.

Despite a wealth of research and development of clinical machine learning tools, few have made it into practice.^5^, ^6^, ^7^ A primary technical concern with the implementation of such models is that their performance can drift over time due to changing populations, measurements, outcome rates, and practices.^8^ Epidemiologists, therefore, argue that a validated prediction model simply does not exist and that their performance should continuously be evaluated.^9^

Even though we have validated our BC stewardship tool in various cohorts in different types of hospitals (e.g., community hospitals, teaching hospitals) and geographical locations (the Netherlands and the United States), it remains at risk of performance drift. Here, we assess the real-time performance of our tool over time in a changing population and the necessity for interventions to optimize its predictions.

## Methods

### Study design and data sources

We performed a real-time evaluation of our BC stewardship tool, which uses the age and sex of the patient, as well as vital signs and regularly measured laboratory values to predict the outcomes of BCs drawn in the ED.^4^ The model has been running in the background (off-policy; not visible to physicians) of our Electronic Health Record (EHR) system in Amsterdam University Medical Centers (UMC; locations VUmc and AMC) between October 2021 and September 2022. During this time, the model predicted the outcome of all BCs drawn from adult patients during their ED stay. Data captured in the EHR system (EPIC Systems Corporation, Verona, Wisconsin, United States) as part of regular care procedures are mapped to the input features of the model. The available data is sent to a separate module of EPIC called Nebula, which hosts the prediction model. Once the model receives sufficient data (at least 20% of the required vital signs and 20% of the laboratory results), it will start returning predicted probabilities for the BC positivity to EPIC every 20 min. The final predictions made during the ED stays are captured in a dashboard, together with the outcome of the BC and the date on which the predictions are performed. This study used those results, which were generated in real time when data was entering the system, supplemented with population characteristics extracted from the EHR. Further details on the development and inner workings of the prediction model can be found in our previous article in *eBiomedicine*.^4^

### Statistics

We conducted an assessment of the model's performance on a monthly basis to identify possible drift over time. The discriminatory performance was assessed using the Area Under the Curve (AUC) of the receiver operating characteristics and the Area Under the Precision-Recall Curve (AUPRC), metrics that were also presented in our initial publication. In addition to these metrics, we examined the Brier score. This score is a well-established metric used to evaluate the precision of probabilistic predictions, and can effectively serve as a substitute for calibration assessments. The Brier score is a bounded measure, ranging from 0 to 1, where a lower score is indicative of a model that yields more accurate predictions. The AUC, AUPRC, and Brier scores were computed in Python (version 3.8.1) using the metric module of the scikit-learn library.

The monthly scores for the AUC, AUPRC, and Brier scores, blood culture outcome rates, and population characteristics were evaluated using Statistical Process Control (SPC) charts for the individuals and moving ranges. These charts can effectively display temporal variation and detect signals of significant changes, which are often indicative of non-random, special cause variations or process drift. The individuals chart shows the individual measurements over time, enabling the identification of any trends, shifts, or cycles that may signify a change in the process. On the other hand, the moving range chart provides insight into the variability of the process by displaying the range between consecutive measurements. The normality of the distribution of the monthly scores was assessed with the Shapiro–Wilk (SW) and the Anderson-Darling (AD) tests. SPC charts were created using the “lolcat” package in R (version 4.2.3).^10^

### Ethics

The local medical ethics review committee of the Amsterdam University Medical Centers (UMC) provided waived the review of this study (IRB number: IRB00002991; case: 2020.486; extension of the previous study), as the Medical Research Involving Human Subjects Act was not deemed applicable. We utilized de-identified data extracts in compliance with the protocol established by our local privacy officer. Consequently, informed consent for data usage was not required.

### Role of funding source

There were no funding sources relating to this work.

## Results

Our BC stewardship tool predicted 3.035 unique adult patient visits with one or more BC draws during the ED stay in Amsterdam UMC (Locations VUmc and AMC) between October 2021 and September 2022. On average, the patients were 65 years old, and 43.1% of the population was female. An overview of the characteristics of the included patients and a comparison with the populations used for training and validating the model previously is presented in Table 1.

**Table 1:** Cohort descriptions of baseline characteristics, predictor variables, and culture outcomes in the datasets used to develop and validate the prediction model for blood culture outcomes.

Variable	VUMC training∗ (n = 6.421)	VUMC test∗ (n = 1.606)	VUmc real-time evaluation (n = 3.035)
Age, median, y (IQR)	66 (52–76)	66 (53–76)	65 (50–75)
Sex, Female, n (%)	3666 (43.2%)	896 (44.2%)	1308 (43.1)
Vital signs, median (IQR)			
Temperature, Celsius	37.7 (36.9–38.5)	37.8 (36.9–38.5)	37.5 (36.6–38.2)
Heart rate,/min	94 (81–106)	93 (81–105)	92 (80–105)
Systolic blood pressure, mmHg	124 (110–140)	123 (110–140)	124 (111–139)
Diastolic blood pressure, mmHg	74 (66–83)	74 (65–83)	74 (66–83)
Respiratory rate,/min	20 (16–25)	20 (16–25)	19 (15–24)
Saturation, %	96 (95–98)	96 (94–98)	97 (95–98)
Laboratory results, median (IQR)			
C-reactive protein	63 (21–141)	58 (19–142)	64 (22–141)
Creatinine	85 (66–119)	84 (65–116)	106 (60–110)
Leukocytes	10.4 (7.0–14.5)	10.3 (6.9–14.5)	9.9 (6.5–13.8)
Outcome			
Positive blood cultures, %	11.5	11.5	13.4
Contaminated cultures, %	6.3	6.3	10.3

The study cohort consists of 3035 unique patient visits in which one or more blood cultures were drawn in the emergency department between October 2021 and September 2022. The training and test set columns were adapter from our previous publication in *eBioMedicine*.^4^

IQR = Interquartile Range; VUMC = VU Medical Center; ∗ = adapted from previous publication.

### Metrics

The average AUC for predicting BC outcomes was 0.78 (standard deviation (SD) = 0.02). The monthly averages ranged between 0.68 and 0.86 and were normally distributed (AD test: p = 0.231; SW test: p = 0.376). The AUPRC average was 0.41 (SD = 0.04), ranged between 0.26 and 0.50, and was normally distributed (AD test: p = 0.458; SW test: p = 0.437). Finally, the Brier score average was 0.10 (SD = 0.01), ranged from 0.076 to 0.114, and was also normally distributed (AD test: p = 0.130; SW test: p = 0.105).

### Blood culture outcomes

During this real-time evaluation, the average BC positivity rate was 13.4% (SD = 1.0%), ranging from 9.2% to 15.6%. The contamination rate average was 10.3% (SD = 1.0%), ranging from 7.4% to 14.6%.

### Population characteristics

As depicted in Table 1, the real-time evaluation cohort demonstrates comparable characteristics to the data employed for model development and testing (also shown in Table 1). Notably, this cohort's blood culture positivity and contamination rates are slightly elevated compared to the previous cohorts. In Table 2, we present diverse outcomes observed in the study population, such as an average hospital admission rate of 63.4%, Intensive Care Unit (ICU) admission rate of 5.7%, a hospital mortality rate of 4.7%, and a 10.8% incidence of COVID-19 diagnoses.

**Table 2:** The outcomes of the real-time evaluation study cohort.

Outcomes	VUmc real-time evaluation (n = 3.035)
ED length of stay, hours, median (IQR)	4.8 (3.5–6.3)
Hospital admission, n (%)	1925 (63.4)
Hospital length of stay, days, median (IQR)	4.6 (2.4–8.2)
ICU admissions, n (%)	174 (5.7)
Hospital mortality, n (%)	143 (4.7)
COVID-19 cases, n (%)	328 (10.8)

The study cohort consists of 3035 unique patient visits in which one or more blood cultures were drawn in the emergency department between October 2021 and September 2022, and in which a prediction by our prediction tool could be made.

ED = Emergency Department; ICU = Intensive Care Unit; VUmc = VU Medical Center.

### Statistical control charts

**Fig. 1:**
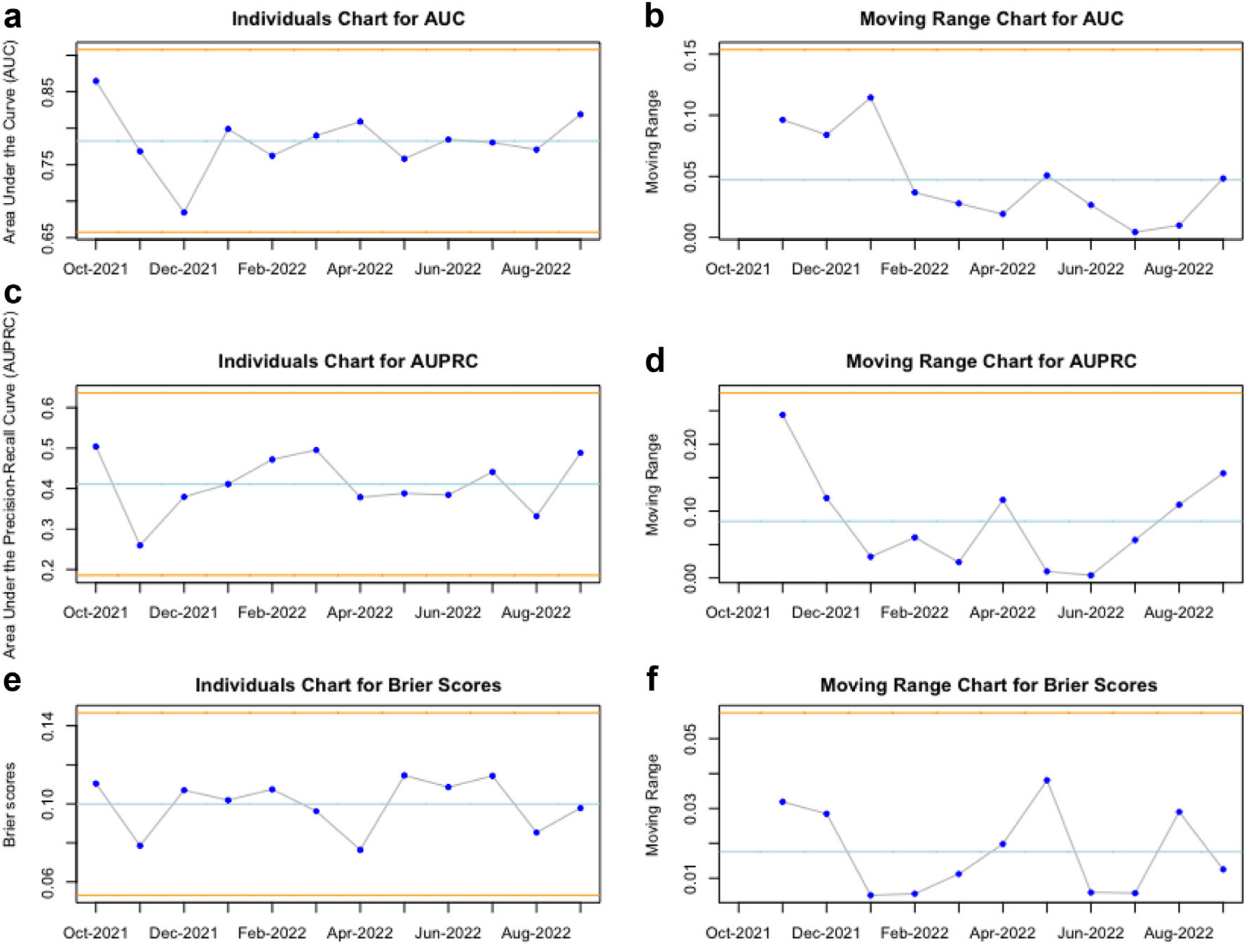
**The statistical process control charts of the individuals and moving range of the average monthly performance of our blood culture prediction tool.** In a cohort of 3.035 unique patient visits to the emergency department, the monthly averages of the area under the curve (AUC), area under the precision-recall curve (AUPRC), and Brier scores were assessed with statistical control charts. The yellow lines in the plots indicate the statistical control range, a function of the mean values and the variation in the data. If any statistical control rules are violated, the data points would be yellow or red. When they are in statistical control, they are blue. A. Shows that the AUC for predicting a positive blood culture remained within control, with an average of 0.78. B. Shows that the moving range of the AUC also remained within control. C. Shows that the AUPRC remained within statistical control, averaging 0.41. D. Shows that the moving range of the AUPRC also remained within control. E. Shows that the Brier score remained within control, averaging 0.10. F. Shows that the moving range of the Brier scores also remained within control.

**Fig. 2:**
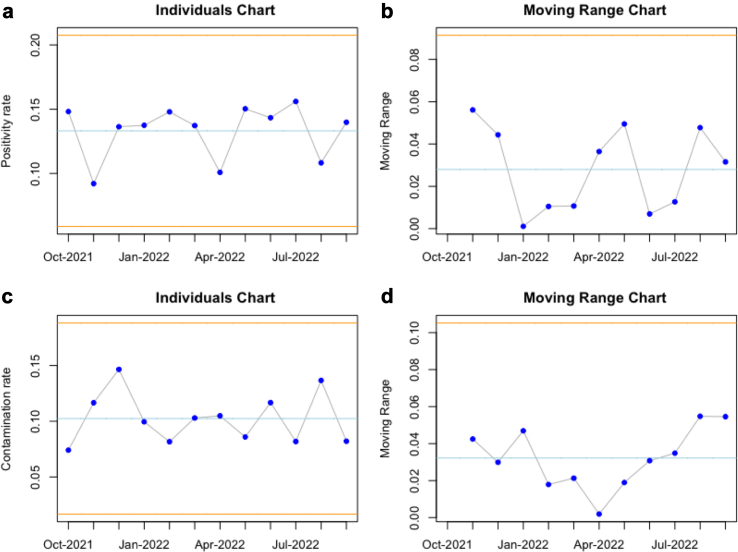
**The statistical process control charts of the individuals and moving range of the blood culture positivity and contamination rates during real-time evaluation.** In a cohort of 3.035 unique patient visits to the emergency department, the monthly averages of the blood culture positivity and contamination rates were assessed with statistical control charts. The yellow lines in the plots indicate the statistical control range, a function of the mean values and the variation in the data. If any statistical control rules are violated, the data points would be yellow or red. When they are in statistical control, they are blue. A. Shows that the blood culture positivity rate remained within control, with an average of 0.134 (13.4%). B. Shows that the moving range of the positivity rate also remained within control. C. Shows that the blood culture contamination rate remained within control, with an average of 0.103 (10.3%). D. Shows that the moving range of the contamination rate also remained within control.

**Fig. 3:**
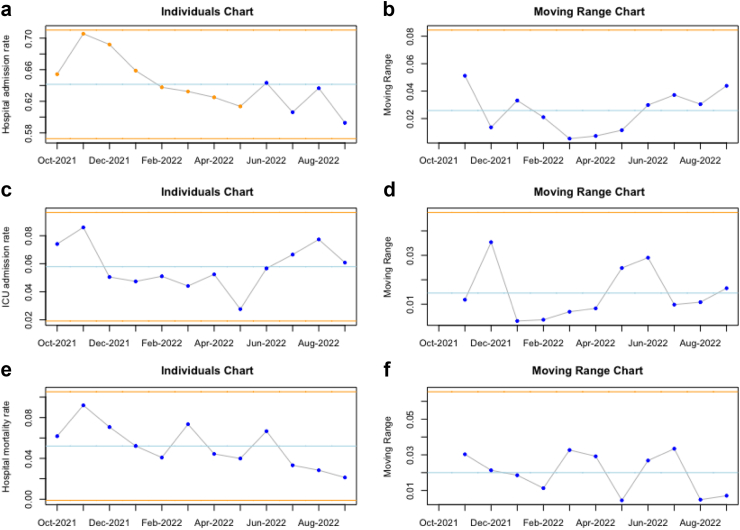
**The statistical process control charts of the individuals and moving range of the average monthly characteristics of the study population.** In a cohort of 3.035 unique patient visits to the emergency department, the monthly averages of the number of daily predictions, hospital admission rates, COVID-19 admission rates, and hospital mortality were assessed with statistical control charts. The yellow lines in the plots indicate the statistical control range, a function of the mean values and the variation in the data. If any statistical control rules are violated, the data points would be yellow or red. When they are in statistical control, they are blue. A. Shows that the average percentage of hospital admissions among the study population decreased over time, outside of statistical control, averaging 0.64%. B. Shows that the moving range of hospital admissions did remain within control. C. Shows that the average percentage of Intensive Care Unit (ICU) admissions remained with in control, averaging 5.8%. D. Shows that the moving range of the COVID-19 admission also remained within control. E. Shows that the hospital mortality rates remained with in control, averaging 0.05. F. Shows that the moving range of the hospital mortality rates also remained within control.

We evaluated the characteristics of the model performance and cohort characteristics over time using SPC charts. The individuals and moving range charts showed no control violations (points outside the statistical control range) over time, which would otherwise have been coloured yellow in the figure for any of the model performance metrics (Fig. 1a–f). Though higher than in the training cohorts, the blood culture positivity and contamination rates also did not show any control violations over time (Fig. 2a–d). Within the population characteristics, the SPC charts showed that the average percentage of hospital admissions within the cohort decreased over time and was outside statistical control (Fig. 3a and b). The ICU admission and hospital mortality rates remained within control (Fig. 3c–f). An updated SPC chart of model metrics until February 2023, made available during this paper's writing, has been added as Supplementary material (see e-Fig. 1 in the Supplementary appendix) and is consistent with the results in Fig. 1a–f.

## Discussion

We evaluated the performance of our BC stewardship tool over time. The average AUC (0.78) during this follow-up is comparable to the original validations (AUCs ranging between 0.75 and 0.81), while the AUPRC (0.41) even is slightly higher (previous AUPRCs ranging between 0.19 and 0.38). The stable Brier Score of 0.10 indicates a well-calibrated model. The BC positivity rate of 13.4% during this study period has been slightly higher than in the original study, just as the contamination rate of 10.3%. Since outcome rates can be significant drivers of performance drifts, this reinforces the need for our efforts to monitor the tool over time. The AUC, AUPRC, and Brier scores showed no statistical control violations over one year, suggesting no performance drift.

Throughout our study, from October 2021 to September 2022, the clinical practice at our center experienced significant transformations related to protocols and patient demographics, primarily due to the COVID-19 pandemic, which was still ongoing at that point. Notably, our population included 328 cases of COVID-19 during the study period (Table 2), even though the prediction model was never trained on data containing COVID-19 patients. Starting in early 2022, our hospital experienced monthly modifications in COVID-19 restrictions and treatment protocols, gradually relaxing their stringency over time. The evolving practices surrounding COVID-19 may thus account for a significant portion of the observed reduction in hospital admission rates, which fell outside the statistical control limits (Fig. 3a). This trend potentially suggests a decrease in disease severity among patients presenting to our emergency department over the specified time frame. Had these shifts in case mix resulted in considerable alterations in model performance, model recalibration could have been employed as a corrective measure.^8^ However, despite evolving practices and populations, the performance of our model consistently remained within the bounds of statistical control, negating the necessity for recalibration and indicating our model is robust to changing environments. The latter is also reinforced by the fact that the initial external validations of our model in various settings (community and teaching hospitals) and locations (the Netherlands and the United States) showed little performance decrease in new environments.

Another potential driver of performance drift can be an alteration in outcome rates, specifically, in our case, the positivity rates of BCs. If there is a change in these rates accompanied by declining model performance, an adjustment to the model's intercept could serve as a correction.^8^ The population in our current study differed significantly from the population on which the model was trained, indicated by the higher blood culture positivity and contamination rates (Table 1). However, throughout our study, no noteworthy deviation in the BC positivity rate was detected beyond the anticipated range of variance. It seems that the model can handle differences in positivity rates. However, we still cannot be sure whether the model performance will remain stable when outcome rates start to vary rapidly and significantly outside of the expected variation.

It is important to note that the use of static machine learning models for predicting time-series data, which can be influenced by changes in the clinical setting, may not possess the same robustness as continual learning methods and continuous retraining. Ethical approval guidelines for trials involving AI algorithms in healthcare and certification guidelines often mandate the use of static models that cannot be modified during evaluation, limiting the use of more robust training methods.^11^ However, as these guidelines evolve and incorporate provisions for continuous retraining, performance drift can be promptly addressed. Nevertheless, the strategies presented in this paper, which allow for tracking performance over time, can still prove valuable in detecting performance declines that may occur despite retraining efforts.

The limited literature on the continuous monitoring of performance drift, as highlighted in the research in context section, implies that this crucial aspect of deploying clinical machine learning models is often neglected. A few studies have investigated model performance monitoring over time, frequently using simulation methods or time-series analyses. Rahmani and colleagues simulated multiple scenarios in which performance drift of their sepsis prediction tool may occur and showed that retraining methods could significantly improve performance afterwards.^12^ Parikh et al. investigated the impact of data shifts related to the COVID-19 pandemic on a mortality prediction algorithm using time-series analyses. They showed that these data shifts led to a substantial decline in predictive performance.^13^ Both approaches may provide some insights into model performances but require complex calculations and may not point to straightforward interventions needed to optimize model performance. Our study presents a simple method to monitor model performance and case mix characteristics, which can provide clear directions on interventions required in case of statistical control deviations. SPC charts can easily be implemented as a dashboard in an EHR system. Minne and colleagues have also suggested this approach toward temporal evaluations by presenting how it helped monitor their classification model for predicting mortality among elderly patients in the ICU.^14^

Several limitations of this study should be acknowledged. Firstly, due to the storage configurations of the EPIC module (Nebula) utilized by our model for predictions, we encountered difficulties retrieving all the necessary information required to characterize the cohort thoroughly. To protect patient privacy, these data points are not stored in the system by default. We retrieved some granular data for this one-year follow-up through different sources available until September 2022. To be as complete as possible, we added the SPC charts of the model performance for the period beyond September 2022 as Supplementary material but could not give insights into the population from that point forward. We are currently awaiting a Nebula reconfiguration that could enable input data storage, providing an even better understanding of the population being tested in future studies and monitoring. The follow-up period of just one year was relatively short, limiting our ability to observe significant changes in case-mix or model performance. Nonetheless, considering the rapid transformations in the healthcare system induced by the COVID-19 pandemic, we have already witnessed substantial population shifts, as indicated by lower admission rates, which did not significantly impact the model's performance. We also acknowledge that our study only captures a fraction of the potential case-mix characteristics that could influence model performance and require further investigation, such as the specific indications used to order BCs. Nevertheless, we can gain valuable insights and obtain a high-level indication of potential drift by monitoring model performance alongside key population characteristics. Moreover, the methods presented here, irrespective of specific changes that may or may not have been detected, offer an effective approach for generating valuable insights that others can use. These methods can be applied to a wide range of metrics relevant to prediction model implementation in the healthcare sector.

Employing techniques to evaluate the performance drift of our machine learning tool over time, we've taken a significant step forward in achieving a safe and effective clinical implementation of the tool. Next, we will need to evaluate how these offline implementations translate to real-world utility. To explore the clinical impact of the tool, we are currently starting a randomized controlled clinical trial, which has been approved by the medical ethics review committee, evaluating whether policies set assisted by AI predictions (intervention) can outperform the decisions made by the physicians alone (control).^15^ During this trial, we will also monitor model performance over time using the SPC charts. While our development paper demonstrated an overall net clinical benefit of using the tool in retrospective data,^4^ it's crucial to consider that the actual clinical impact might diverge due to factors like non-adherence or the varying effects of false positive or negative alerts.

Taken together, we showcased a simple and effective way of tracking the performance of a machine learning model over time. Using SPC charts for various metrics enables proper monitoring of potential performance drift. Simultaneous assessment of the variation of outcome rates and population changes can guide the specific interventions, such as intercept correction or recalibration, needed to maintain a stable model performance over time. While a truly validated prediction may simply not exist, monitoring will keep us close to the desired standard.^9^ This real-time evaluation of ED patients undergoing BC draws suggests that our model's performance is stable and does not need recalibration or correction yet.

## Contributors

MS, WJW, and PWBN conceptualized this study. MS, AWB, and KP curated the data. MS, AWB, KP, WJW, and PWBN collectively investigated the data and decided on the methodology. MS conducted the formal analyses. AWB, KP, WJW, and PWBN supervised various parts of the research process within their expertise. MS, AWB, KP, WJW, and PWBN acquired funding. MS, AWB, and KP drafted the original manuscript. MS and AWB accessed and verified the underlying data. MS, AWB, KP, WJW, and PWBN reviewed, edited, and agreed with the final version of the manuscript.

## Data sharing statement

The data underpinning this study is available for sharing. Following the publication of this work, requests for data can be made. Researchers who present a methodologically robust proposal compliant with our local privacy regulations can access the data. Requests should be directed towards the corresponding author, and a signed data access agreement will be required.

## Declaration of generative AI and AI-assisted technologies in the writing process

During the preparation of this work the author(s) used ChatGPT and Grammarly in order to rephrase some of the sentences for clarity. After using this tool/service, the author(s) reviewed and edited the content as needed and take(s) full responsibility for the content of the publication.

## Declaration of interests

The authors declare no competing interests regarding this work.
