# Supplementary material for: Detecting changes in the performance of a clinical machine learning tool over time
Source: eBioMedicine. 2023 Oct 2;97:104823. doi: 10.1016/j.ebiom.2023.104823 (PMC10550508; doi:10.1016/j.ebiom.2023.104823)

# Supplementary appendix

**e-Figure 1. The statistical process control charts of the individuals and moving range of the average monthly performance of our blood culture prediction tool.**

In a cohort of 4.771 unique patient visits to the emergency department between October 2021 and February 2023, the monthly averages of the area under the curve (AUC), area under the precision-recall curve (AUPRC), and Brier scores were assessed with statistical control charts. The yellow lines in the plots indicate the statistical control range, a function of the mean values and the variation in the data. If any statistical control rules are violated, the data points would be yellow or red. When they are in statistical control, they are blue. A. shows that the AUC for predicting a positive blood culture remained within control, with an average of 0.77. B. shows that the moving range of the AUC also remained within control. C. shows that the AUPRC remained within statistical control, averaging 0.41. D. shows that the moving range of the AUPRC also remained within control. E. shows that the Brier score remained with in control, averaging 0.10. F. shows that the moving range of the Brier scores also remained within control.

^
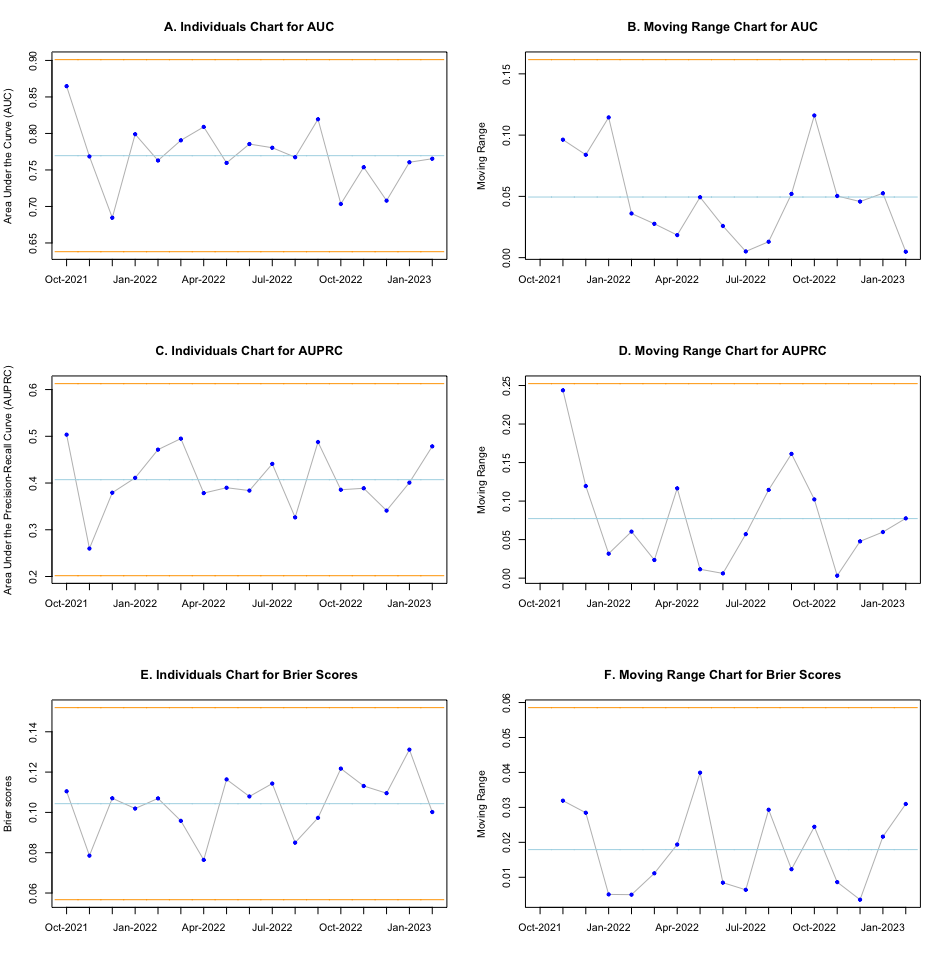
^

**e-Figure 2. The statistical process control charts of the individuals and moving range of the blood culture positivity rates during real-time evaluation.**

In a cohort of 4.771 unique patient visits to the emergency department between October 2021 and February 2023, the monthly averages of the blood culture positivity rates were assessed with statistical control charts. The yellow lines in the plots indicate the statistical control range, a function of the mean values and the variation in the data. If any statistical control rules are violated, the data points would be yellow or red. When they are in statistical control, they are blue. A. shows that the blood culture positivity rate remained within control, with an average of 0.139 (13.9%). B. shows that the moving range of the positivity rate also remained within control.


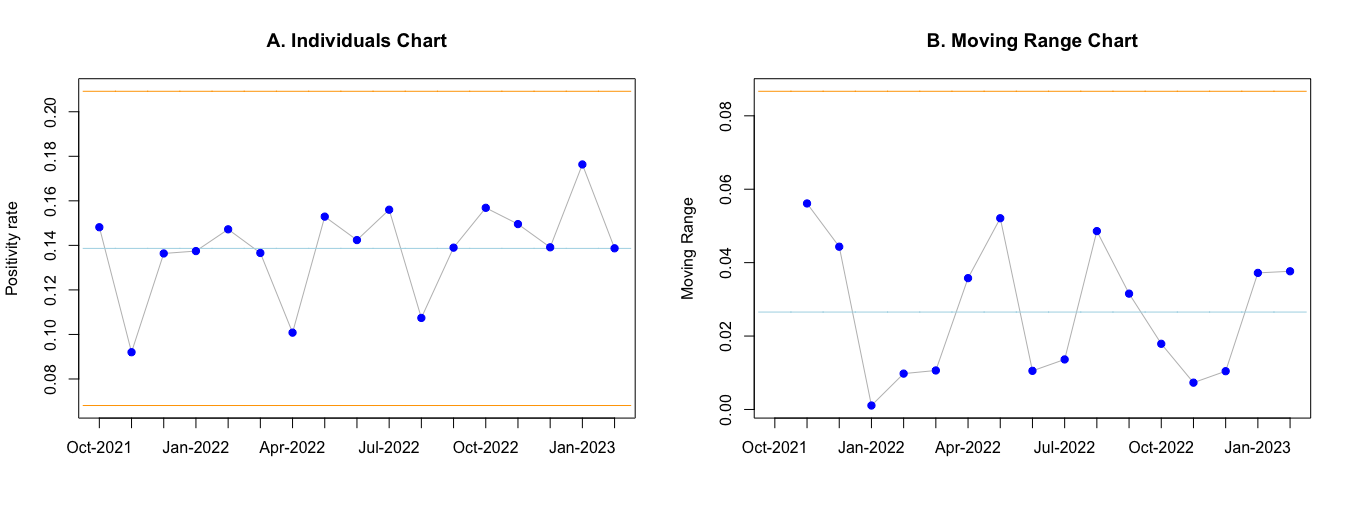

Supplement: Supplementary appendix [file mmc1.docx]
